# Stromal–Epithelial Interactions in Cancer Progression and Therapy Response

**DOI:** 10.3390/cancers15113014

**Published:** 2023-06-01

**Authors:** Manish Thiruvalluvan, Neil A. Bhowmick

**Affiliations:** Department of Medicine, Cedars-Sinai Medical Center, Los Angeles, CA 90048, USA; manish.thiruvalluvan@cshs.org

Tumorigenesis is a result of cell-intrinsic epigenomic and genomic changes as well as cell-extrinsic factors. It has been recognized for some time that cancer cells are not alone, but coexist alongside a variety of extracellular components in the tumor microenvironment (TME). When chemotherapy or targeted therapy, respectively, inhibit proliferation or restrict oncogenic dependence of cancer cells, the TME comprised of, fibroblasts, extracellular matrix, immune cells, vasculature and exosomes are also impacted. Often, these changes elicited by the therapy or the tumor response to the therapy contribute to the eventual development of drug resistance [1,2,3,4,5]. In this Special Issue of *Cancers*, the focus is on the role of stromal–epithelial interactions as drivers of tumorigenesis as well as the co-evolution of the tumor and its microenvironment as contributors of therapy resistance.

Cell extrinsic factors can serve as biomarkers and therapeutic targets. One such source of biomarkers includes tumor-derived circulating tumor DNA (ctDNA). Emerging data have shown that ctDNA can be an effective biomarker for the early detection of various cancers from breast to colon cancer, to predict the likelihood of cancer recurrence and inform what form of treatment is most effective in improving patient mortality, reviewed by Gong et al. [6]. CtDNA as well as exosomal cargo of nucleic acids and proteins are demonstrated robust biomarkers in many cancer types. Borgmann et al. reviewed the role of immunosurveillance disruption in the development of esophageal adenocarcinoma (EAC) [7]. Immune tolerance is found to be perpetuated by the chronic inflammatory environment inherent to Barret’s esophagus. Although the mechanism of immune tolerance is still being discovered, there are opportunities to use known mediators as biomarkers of early detection and potentially even as therapeutic targets.

Many metabolites are present in the TME that have dual roles in metabolism and cell signaling. One such metabolite is glutamine, which has been established as a conditionally essential amino acid in cancer cell metabolism. Thiruvalluvan et al. showed that prostate cancer tumors express elevated glutamine transporters (e.g., SLC1A5 and SLC38A1) compared to their benign counterparts [8]. Considering glutaminase inhibitors have been used before to limited clinical benefit, they hypothesized that directly depleting glutamine in the extracellular space may be more prudent in reducing tumor growth. To this end, they introduced L-asparaginase to hydrolyze glutamine into glutamate as a way to increase radiosensitivity in prostate cancer cells. Previous studies demonstrated that CAF-derived glutamine contributed hormone therapy resistance [2]. They demonstrated that removing glutamine in this way potentiates radiation-induced cellular toxicity through the induction of ER stress. Prostate cancer tissue recombination models non-responsive to irradiation were sensitized by asparaginase administration [8]. This highlighted the critical role stromal fibroblasts play in supplying epithelial cells with vital nutrients to avoid cellular toxicity and that targeting both epithelial and stromal compartments can lead to better therapeutic outcomes.

Kakarla et al. demonstrated the role of Ephrin ligand expression in epithelia to transform surrounding stroma in CAFs [9]. Ephrin signaling is a prominent example of juxtacrin signaling often disrupted in tumor progression. Fibroblasts gathered from the peripheral zone of prostate tumors had increased expression of EFNB2 and EFNB3 when compared to those from the transition zone. Then, they demonstrated that continuous activation of EFNB1 and EFNB3 in vitro dramatically increased the expression of CAF markers such as alpha-smooth muscle actin (α-SMA) and tenascin-C (TNC) through the activation of Src family kinases in stromal cells. The Src-mediated acquisition of CAF features was a result of elevated EFNB ligands found in prostate cancer TME [9]. An Src inhibitor, Saracatinib (AZD0530), dramatically lowered the phosphorylation of Src targets and effectively reduced α-SMA and TNC expression by fibroblasts.

Extracellular vesicles, exosomes, have come to the forefront in the TME vernacular, recognized in recent years as key mediators of cancer progression and metastasis [10,11]. Patel et al. focused on elucidating the role of microRNAs (miRs), as exosomal cargo, in the potentiating prostate cancer transdifferentiation to a neuroendocrine phenotype [12]. Numerous studies have shown the transfer of miRs between cells via exosomes as a means of communication. Neuroendocrine prostate cancer, while a rare event de novo is a well-described development in response to hormone therapy, contributing to therapy resistance [13]. Disrupting this signaling axis via miRs may be key to helping patients with end-stage prostate cancer. In this paper, the authors demonstrated the role of TBX2 in NEPC, previously established as a transcription factor having a key role in prostate cancer bone metastasis [14]. TBX2-mediated repression of miR-200c-3p caused an increase in SOX2 and N-MYC, leading to greater phenotypic plasticity. They showed that stable expression of miR-200c-3p in deficient cell types is enough to reverse numerous NEPC markers as well as decrease the expression of SOX2 and N-MYC [12]. The work highlighted the impact that cancer-derived factors can have in an autocrine and paracrine manner to influence the differentiation state and therapy responsivity of the tumor.
